# Suppressing Effect of Baclofen on Multiple Alcohol-Related Behaviors in Laboratory Animals

**DOI:** 10.3389/fpsyt.2018.00475

**Published:** 2018-09-28

**Authors:** Giancarlo Colombo, Gian Luigi Gessa

**Affiliations:** Neuroscience Institute, Section of Cagliari, National Research Council of Italy, Monserrato, Italy

**Keywords:** baclofen, GABA_**B**_ receptor, alcohol drinking and seeking, reinstatement of alcohol seeking, animal models of alcohol use disorder

## Abstract

This paper summarizes the several lines of experimental evidence demonstrating the ability of the prototypic GABA_B_ receptor agonist, baclofen, to suppress multiple alcohol-related behaviors in laboratory rodents and non-human primates exposed to validated experimental models of alcohol use disorder (AUD). Specifically, treatment with baclofen has repeatedly been reported to suppress alcohol-induced locomotor stimulation, alcohol drinking (including binge- and relapse-like drinking), operant oral alcohol self-administration, alcohol seeking, and reinstatement of alcohol seeking in rats and mice. Treatment with baclofen also reduced operant oral alcohol self-administration in baboons. Several of these effects appear to be mediated by GABA_B_ receptors located in the ventral tegmental area. The often observed co-occurrence of “desired” pharmacological effects and “unwanted” sedative effects represents the major drawback of the preclinical, *anti*-alcohol profile of baclofen. Collectively, these data underline the role of the GABA_B_ receptor in the mediation of several alcohol-related behaviors. These data possess remarkable translational value, as most of the above effects of baclofen have ultimately been reproduced in AUD patients.

## Baclofen effect on alcohol-induced stimulation of locomotor activity

Historically, the first study investigating the effect of the GABA_B_ receptor orthosteric agonist, baclofen, on an alcohol-induced response *in vivo* was conducted in Sweden more than 40 years ago (1); it included, amongst the authors, the Nobel laureate Arvid Carlsson. This study found that acute pretreatment with 5 mg/kg baclofen (i.p.) completely abolished alcohol-stimulated locomotor activity in mice. Besides being the “foundation stone” in the alcohol-baclofen research field, this study was of relevance for the translational value of its data: drug-induced hyperlocomotion in rodents and euphoria in humans are indeed homologous phenomena, as they are mediated by common neural systems [see (2)]; accordingly, in their paper Carlsson and his colleagues wrote that their finding “may be of heuristic value in the treatment of chronic alcoholism,”, hypothesizing the translation of the ability of baclofen to suppress alcohol-stimulated locomotor activity in mice to alcohol stimulating and euphorigenic effects in humans.

These initial results have been confirmed by a series of subsequent studies reporting the suppressing effect of acutely administered baclofen on alcohol-stimulated locomotor activity in alcohol-preferring “University of Chile bibulous” (UChB) rats (3) and mice belonging to different strains and lines (4–9). Baclofen-induced suppression of alcohol-stimulated locomotor activity has also been recorded in preweanling (12-day-old) Sprague-Dawley rats (10), suggesting the involvement of the GABA_B_ receptor system in the mediation of the stimulating effects of alcohol already in this developmental period.

## Baclofen effect on alcohol drinking

### Homecage alcohol drinking

Human alcohol drinking is conventionally modeled in rodents by exposing rats and mice—usually singly housed in their homecage—to the choice between a water bottle and a bottle containing an alcohol solution. Under this simple procedure, animals can freely choose when and how much alcohol they drink. Under specific experimental conditions, this regimen results in voluntary intakes of psychopharmacologically relevant amounts of alcohol, mimicking excessive alcohol drinking (*too much, too often*) in humans and providing a reliable tool for pharmacological tests [see (11)].

Several studies have used the 2-bottle “alcohol vs. water” choice regimen to investigate baclofen effect on alcohol drinking. In most studies, acute or repeated systemic administration of baclofen produced substantial reductions in alcohol intake [(3, 12–16); see however (17)] in alcohol-experienced rats and mice. “Alcohol-experienced” means that, in these animals, consumption of high doses of alcohol was already consolidated before the start of treatment with baclofen; these animals are thought to model the “maintenance” or “active drinking” phase of human alcohol use disorder (AUD). Repeated i.p. treatment with 2.5 and 5 mg/kg racemic baclofen or the corresponding doses of R(+)-baclofen (the active enantioner) markedly reduced daily alcohol intake in selectively bred Sardinian alcohol-preferring (sP) (13) and alcohol-preferring UChB (3) rats; fully compensatory increases in daily water intake and lack of effect on spontaneous locomotor activity indicated that the reducing effect of baclofen on alcohol intake was not attributable to any baclofen-induced motor-incapacitating or sedative effect (that would otherwise disrupt the normal rates of drinking) (3, 13).

Although generally consistent in reporting a reducing effect of treatment with baclofen on alcohol drinking, studies conducted to date have reported considerable differences in baclofen efficacy. These differences are likely due to diversities in drinking history (resulting or not in the development of an addiction-like state) and genetic background of rats and mice used in those studies. An additional explanation of the differential efficacy of baclofen in reducing alcohol drinking has been proposed to reside in the at times reported opposite effect of R(+)- and S(-)-baclofen enantiomers on some alcohol-related behaviors [(18); see however (19)].

The suppressing effect of 5 mg/kg baclofen (i.p.) on alcohol intake in rats was fully prevented by pretreatment with the GABA_B_ receptor antagonists, phaclofen and CGP36742 (20). These results provide strong evidence that baclofen effect on alcohol drinking is secondary to activation of the GABA_B_ receptor.

Repeated treatment with baclofen (1–3 mg/kg, i.p.) over the initial phase of exposure to alcohol prevented acquisition of alcohol drinking in sP rats (21). These data suggest that activation of the GABA_B_ receptor blocked detection of the psychopharmacological effects of alcohol underlying alcohol drinking. Rats acquired alcohol drinking behavior only once treatment with baclofen had been ceased (21). The translational value of these results may reside in pharmacologically delaying the onset of excessive alcohol drinking during adolescence and early adulthood, with the intent of potentially reducing the risk of developing AUD later in life.

### Binge-like drinking

Baclofen has also been tested in two validated mouse models of binge drinking, named “drinking-in-the-dark” (DID) and “scheduled high alcohol consumption” (SHAC), respectively. The DID procedure is based on exposure of mice to alcohol in brief (2–4 h) daily drinking sessions occurring at fixed times during the early period of the dark phase of the daily light/dark cycle [(22); for review, see (23)]. In the SHAC procedure, water-deprived mice are given daily periods of fluid access varying between 4 and 10 h; every third day, an alcohol bottle is presented over the initial 30 min of the drinking session (24). Both procedures result in excessive alcohol drinking and intoxicating blood alcohol levels at the end of the drinking session, reproducing the “*too much, too fast*” condition of human binge drinking.

In the “DID” studies, systemic administration of doses of R(+)-baclofen in the 5–10 mg/kg range markedly reduced alcohol intake in C57BL/6J mice (18, 25), selectively bred High Alcohol Preferring 1 (HAP1) mice (18), and High DID (HDID) mice (selectively bred for high alcohol drinking under the DID regimen) (26). When tested in the SHAC procedure, baclofen (2.5 and 5 mg/kg, i.p.) dose-dependently reduced—up to complete suppression—alcohol intake in Withdrawal Seizure Control mice (27).

### Relapse-like drinking

Relapse drinking is modeled in rodents by the so-called “alcohol deprivation effect” (ADE), that is the transient increase in voluntary alcohol intake occurring after a relatively long period of forced abstinence from alcohol [see (28)]. The effect of baclofen on ADE has been investigated in alcohol-preferring sP rats (29, 30) and alcohol-addicted Wistar rats (31).

In the two studies employing sP rats (29, 30), animals were initially exposed to a period of continuous alcohol drinking under the 2- or 4-bottle “alcohol vs. water” choice regimen (in the latter case, multiple alcohol concentrations were concurrently available), during which rats developed high and steady daily intakes of alcohol. Rats were then deprived of alcohol for 7–14 consecutive days. Re-exposure to alcohol resulted in a substantial increase in alcohol intake over the first hour of re-access as well as in a shift in preference for the highest concentrated alcohol solution. Acute treatment with baclofen (1–3 mg/kg, i.p.), administered immediately before re-exposure to alcohol, produced a complete blockade of both ADE-associated events (increase in alcohol intake and shift in alcohol preference). The lack of baclofen effect on food and water intake and spontaneous locomotor activity conferred specificity for alcohol intake to the reducing effect of baclofen (29, 30).

In the study employing Wistar rats (31), animals were exposed to long periods of alcohol drinking interposed with periods of alcohol abstinence, resulting—after ~1 year—in the development of addiction-like behaviors. Baclofen (1 and 3 mg/kg, i.p.) was injected repeatedly over the last drinking and deprivation phases. Treatment with 3, but not 1, mg/kg baclofen (i.p.) significantly reduced the post-abstinence, extra-intake of alcohol; specificity was not complete, as the reducing effect of baclofen on alcohol drinking was accompanied by a reduction in spontaneous locomotor activity, suggestive of a sedative effect. Again, drinking history and genetic background of rats may explain the differences observed in the ADE studies on baclofen.

## Baclofen effect on operant alcohol self-administration

A major step forward in the assessment of the preclinical, *anti*-alcohol profile of baclofen has been represented by the several studies using operant procedures of oral alcohol self-administration, in which alcohol is made available once the animal has activated an *operandum* (usually a lever or a nose-poke). Operant procedures allow measurement not only of the mere consumption of alcohol but also of the amount of “work” that the animal is willing to perform to access alcohol; when a high workload is required, operant procedures well model the human condition of excessive amounts of time spent in obtaining and using alcohol [a fundamental criterion for diagnosis of AUD (32)].

Rodent studies on baclofen have focused mainly on the use of two different procedures of oral alcohol self-administration: (i) fixed ratio (FR) schedule of reinforcement and (ii) progressive ratio (PR) schedule of reinforcement. Under the FR schedule, response requirement (RR; i.e., the “cost” of each alcohol presentation in terms of responses on the *operandum*) is predetermined and maintained throughout the session; this schedule provides a measure of alcohol consumption and reinforcing properties of alcohol [see (33)]. Under the PR schedule, RR is progressively increased after the delivery of each reinforcer; the lowest ratio not completed (breakpoint) provides a measure of motivational properties of alcohol [see (33)].

Rat studies have almost unequivocally reported the ability of systemic baclofen [0.5–5.6 mg/kg (i.p.) or corresponding doses of R(+)-baclofen] to markedly reduce alcohol self-administration under the FR schedule of reinforcement (with FR varying from FR1 to FR4); this effect was observed both in selectively bred alcohol-preferring rats (19, 34–36) and unselected Wistar and Long Evans rats (37–42). A mouse study reported similar results: treatment with baclofen (1–17 mg/kg, i.p.) reduced lever-responding for alcohol (43).

Interesting results, with possible translational value, have been yielded by “FR” studies testing baclofen in rats with high levels of responding for alcohol and large amounts of self-administered alcohol. Specifically, treatment with baclofen resulted to be particularly effective in Indiana alcohol-preferring (P) rats (36) and Wistar rats made physically dependent on alcohol by exposure to alcohol vapors (40). These data apparently mirror the results of clinical studies suggesting a greater efficacy of baclofen in patients affected by severe AUD [see (44)].

The relatively few studies using the PR schedule of reinforcement generated results similar to those of the “FR” studies: treatment with baclofen (1–3 mg/kg, i.p.) effectively reduced lever-responding and breakpoint for alcohol in different lines of selectively bred alcohol-preferring rats (36, 45) as well as in alcohol-dependent and –non-dependent Wistar rats (40).

The selectivity of the reducing effect of baclofen on alcohol self-administration was limited; treatment with baclofen reduced indeed self-administration of alternative, non-drug reinforcers—such as sucrose or saccharin solutions or regular food pellets—with potency and efficacy often comparable to those observed in “alcohol” experiments (34, 36, 38, 39, 45). Some studies also reported partial overlap between baclofen doses reducing alcohol self-administration and those inducing hypolocomotion (43), suggesting a possible confounding impact of baclofen-induced motor-incoordination on lever-responding for alcohol.

Operant procedures also allow the investigation of alcohol seeking, separated from any alcohol drinking. This may be done with animals trained to respond for alcohol and then exposed to test sessions during which responding are never reinforced: the only measurable outcome is extinction responding (ER). ER is taken as measure of the appetitive and motivational properties of alcohol [see (33)]. An alternative experimental procedure (commonly named “sipper”) – that provides a clear separation between the appetitive and consummatory phases of alcohol self-administration within a single session—is based on animals trained to complete a single RR (usually, 16–30 lever-responses) to gain access to alcohol for a substantial period of time (usually, 20 min) [see (46)]. In an “ER” study (47), treatment with 1–3 mg/kg baclofen (i.p.) completely suppressed ER for alcohol in alcohol-preferring sP rats; although not influenced by any motor-incoordinating effect, the suppressing effect of baclofen on ER was not selective for alcohol, as baclofen treatment also suppressed ER for a sucrose solution. A subsequent “ER” study using Long Evans rats reported a comparable reduction of ER for alcohol and sucrose after treatment with 3 mg/kg baclofen (i.p.) (48). When tested under the “sipper” procedure, systemic administration of baclofen reduced lever-responding to access alcohol in Long Evans rats (48) and C576BL/6 mice (27); in both studies, treatment with baclofen also affected lever-responding for sucrose, limiting the selectivity of baclofen effect.

Two recent studies (49, 50) investigated the effect of baclofen on alcohol self-administration in non-human primates. In both studies, adult male baboons were exposed to a chained schedule of reinforcement with three linked components; sequential completion of RR of each component made alcohol available. Baclofen was injected intramuscularly at doses varying between 0.1 and 2.4 mg/kg. Treatment with baclofen decreased lever-responding for alcohol, number of alcohol drinks, and amount of self-administered alcohol. Treatment with baclofen was effective when initiated during ongoing alcohol access, but not when initiated during an alcohol-abstinence period preceding alcohol access (50). These data are in agreement with the results of human studies indicating a greater efficacy of baclofen when treatment was initiated during active drinking [e.g., (51)] rather than after abstinence had been achieved [e.g., (52)]. An additional experiment found that baclofen treatment facilitated ER for alcohol (49). As in the majority of rodent studies of alcohol self-administration, in these two monkey studies selectivity of baclofen effect was modest, as baclofen also reduced self-administration of a non-drug reinforcer (orange-flavored beverage). Sedation and transient side-effects were also observed.

## Baclofen effect on reinstatement of alcohol seeking

Two rodent studies investigated the effect of baclofen on reinstatement of alcohol-seeking behavior. In this procedure, largely used in the alcohol and drug addiction field, previously extinguished unreinforced lever-responding or nose-poking for alcohol is reinstated by (i) environmental stimuli previously associated to alcohol availability, (ii) limited availability of alcohol, (iii) exposure to stressors, or (iv) administration of specific drugs (e.g., nicotine and cannabinoids). Reinstatement of alcohol-seeking behavior models loss of control over alcohol and relapse episodes in AUD patients [see (28)], resulting to be complementary to ADE.

In the first study (53), alcohol-preferring sP rats—trained to lever-respond for alcohol under the FR4 schedule of reinforcement—were exposed to a single session made of an initial phase of ER followed, once lever-responding for alcohol was virtually completely extinguished, by a reinstatement phase; the latter was preceded by an alcohol-associated stimulus complex, that effectively reinstated lever-responding (still unreinforced). Acute treatment with baclofen (3 mg/kg, i.p.), given after extinction of lever-responding and 30 min before presentation of the alcohol-associated stimulus complex, resulted in a marked reduction of an otherwise robust reinstatement of lever-responding. These data complement well with those reporting the ability of 1–3 mg/kg baclofen in suppressing relapse-like drinking in sP rats exposed to the ADE procedure (29, 30).

In the second study (31), Wistar rats—trained to nose-poke for alcohol under the FR3 schedule of reinforcement—were first exposed to a series of ER sessions and then to reinstatement sessions in which unreinforced nose-poking was reinstated by the presentation of alcohol-associated stimuli. Treatment with baclofen (1 and 3 mg/kg, i.p.) fully abolished, in a dose-related manner, reinstatement of nose-poking.

## Mechanism of action of baclofen effects on alcohol-related behaviors

Several lines of experimental evidence converge to support the hypothesis that the suppressing effect of baclofen on multiple alcohol-related behaviors are mediated by the activation of GABA_B_ receptors located in the ventral tegmental area (VTA), a key area of the mesolimbic dopamine system [i.e., the neuronal system mediating the rewarding, reinforcing, motivational, and stimulating properties of natural stimuli and drugs of abuse [see (54)], including alcohol [see (55)]. Indeed, baclofen microinjection into the VTA of rats and mice suppressed several alcohol-motivated behaviors and *in vivo* actions of alcohol, including (a) alcohol drinking (56), (b) alcohol self-administration (57), (c) alcohol seeking (58), (d) alcohol-stimulated locomotor activity (8, 59), (e) alcohol-induced sensitization of locomotor activity (59), and (f) alcohol-induced conditioned place preference (60). More specifically, intra-VTA microinjection of 0.03–0.3 μg baclofen dose-dependently suppressed lever-responding for alcohol in sP rats (57); suppression of lever-responding for alcohol was particularly evident over the first minutes of the test session, suggestive of the ability of intra-VTA baclofen to suppress rat motivation to start seeking and consuming alcohol. The effect of intra-VTA baclofen on alcohol self-administration was site-specific and devoid of any motor-incoordinating effect (57).

In close agreement with the above data, and as further confirmation of the hypothesis relating to the hyperpolarization of mesolimbic dopamine neurons as the mechanism of action through which baclofen suppresses alcohol-related behaviors, intra-VTA microinjection of baclofen suppressed dopamine release—stimulated by cues anticipating alcohol reinforcement—in the rat nucleus accumbens (NAc; the target brain area of mesolimbic dopamine neurons) (58, 61, 62).

Brain regions other than VTA likely contribute to mediation of the suppressing effects of baclofen on alcohol-related behaviors. Accordingly, recent experimental data highlighted a role for the ventral pallidum (VP), a brain area to which GABAergic neurons located in the NAc project their axons; indeed, baclofen microinjection into the VP reduced alcohol intake in selectively bred alcohol-preferring Alko Alcohol (AA) rats (63). More recent data pointed to involvement of the amygdala: due to reduced levels of the GABA transporter GAT3, high concentrations of extracellular GABA have been found in the amygdala of alcohol-dependent rats (64). It has been proposed that baclofen-induced activation of amygdalar presynaptic GABA_B_ receptor would lower extracellular GABA, possibly normalizing the enhanced tonic inhibition of amygdala and, subsequently, reducing excessive alcohol drinking (65).

## Baclofen effect on alcohol withdrawal syndrome

An additional series of *in vivo* studies tested the ability of baclofen to ameliorate signs of alcohol withdrawal syndrome (AWS) in rats. AWS is experimentally induced by the abrupt termination of alcohol administration to rats made physically dependent on alcohol by the forced, prolonged exposure to intoxicating amounts of alcohol [e.g., (66)]. The resulting signs closely resemble those observed in AUD patients (67, 68).

Treatment with baclofen (1.25–25 mg/kg, i.p.) suppressed several AWS signs, including increase in alcohol self-administration, anxiety-related behaviors, tremors, and seizures in alcohol-dependent Wistar, Sprague-Dawley, and Lister rats (13, 40, 69, 70). The ability of baclofen to effectively suppress AWS signs in rats has since been effectively translated to AUD patients [e.g., (71)]. A GABA_B_ receptor-mediated counterbalance of the AWS-associated, enhanced function of glutamate excitatory neurotransmission has been proposed as the mechanism by means of which baclofen exerts its suppressing effects on AWS signs (13, 40).

## Conclusions

Treatment with the prototypic, orthosteric agonist of the GABA_B_ receptor, baclofen, suppressed several different alcohol-related behaviors in laboratory rodents and non-human primates. These results are of relevance as they suggest a critical role for the GABA_B_ receptor in the neural substrate underlying alcohol seeking and drinking. Additionally, these data possess remarkable translational value, as most of the effects of baclofen observed in animals have subsequently been reproduced in AUD patients: most of the clinical surveys conducted to date indeed reported substantial reductions in alcohol drinking and craving for alcohol after treatment with baclofen [see (62, 72)], making baclofen a promising pharmacotherapy for AUD.

The limited separation between baclofen-induced “desired” pharmacological effects and “unwanted” sedative effects appears to be the most relevant drawback of the *anti*-alcohol profile of baclofen. A major step forward in this field is likely represented by the positive allosteric modulators (PAMs) of the GABA_B_ receptor; notably, rodent data collected to date on this new class of GABA_B_ receptor ligands suggest indeed that GABA_B_ PAMs reproduce all the *anti*-addictive properties of baclofen at doses largely lower than those inducing motor-incoordination and sedation [see (62, 73–75)]. Since the first GABA_B_ PAMs are now approaching clinical testing, it will soon be possible to assess whether they retain baclofen effects, while possessing higher therapeutic index and more favorable side-effect profile, also in humans.

## Author contributions

GC and GG contributed equally to literature search and manuscript writing. Both authors approved the final draft of the manuscript.

### Conflict of interest statement

GC and GG are inventors of a European patent on “The use of baclofen in the treatment of alcoholism”. GC is partner of Cagliari Pharmacological Research (Cagliari, Italy), owner of the above patent.
